# Supplementary material for: Optimization of Laccase from Ganoderma lucidum Decolorizing Remazol Brilliant Blue R and Glac1 as Main Laccase-Contributing Gene
Source: Molecules. 2019 Oct 30;24(21):3914. doi: 10.3390/molecules24213914 (PMC6864837; doi:10.3390/molecules24213914)
Supplement: Supplementary file 1 [file molecules-24-03914-s001.pdf]

Supplementary:

This supplementary document is the relative expression levels of other nine *G. Lucidum* laccase genes under RBBR, which serves for the result "3.5 Transcriptional analysis of laccase genes" in article.

Table S1 Relative expression levels of other nine *G. Lucidum* laccase genes under RBBR.

Time	*Glac2*	*Glac3*	*Glac6*	*Glac7*	*Glac8*	*Glac9*	*Glac13*	*Glac14*	*Glac15*
0 h	0.16	2.86	15.28	11.36	2.76	3.99	0.06	1.64	2.26
1 h	0.10	0	28.41	80.34	12.02	47.69	6.10	7.80	2.41
5 h	0.23	0	0.07	0.08	0.02	0.06	0	0.01	0
10 h	0.59	0	0.84	0.30	0.28	0.31	0.01	0.06	0.01

Table S2 GenBank accession number of Glac genes

Gene name	accession numbers	Gene name	accession numbers	Gene name	accession numbers
Glac1	KC507935	Glac6	KC5079340	Glac11	KC5079345
Glac2	KC507936	Glac7	KC5079341	Glac12	KC5079346
Glac3	KC507937	Glac8	KC5079342	Glac13	KC5079347
Glac4	KC507938	Glac9	KC5079343	Glac14	KC50793 48
Glac5	KC507939	Glac10	KC5079344	Glac15	KC5079349
